# Trauma and posttraumatic growth in cancer: validation of the post-traumatic stress disorder checklist for DSM-5 and the posttraumatic growth inventory-short form in patients with breast cancer

**DOI:** 10.3389/fpsyt.2026.1855703

**Published:** 2026-10-20

**Authors:** Cláudio Castanheira, Sílvia Almeida, Isabel Manica, André Abreu, Diana Frasquilho, Berta Sousa, Maria Vânia Silva-Nunes, Albino J. Oliveira-Maia, Raquel Lemos

**Affiliations:** 1 Champalimaud Clinical Centre, Champalimaud Foundation, Lisbon, Portugal; 2 Faculdade de Ciências da Saúde e Enfermagem, Universidade Católica Portuguesa, Lisbon, Portugal; 3 Graduate Programme in Clinical and Health Psychology, Faculdade de Psicologia da Universidade de Lisboa, Lisbon, Portugal; 4 Digital Neurotherapeutics Centre, Champalimaud Foundation, Lisbon, Portugal; 5 Centro de Investigação Interdisciplinar em Saúde (CIIS), Universidade Católica Portuguesa, Lisbon, Portugal; 6 Clinical and Health Services Research, Faculty of Medicine of the University of Porto (FMUP), Porto, Portugal; 7 Neuroscience of Disease Programme, Champalimaud Foundation, Lisbon, Portugal; 8 NOVA Medical School (NMS), Faculdade de Ciências Médicas (FCM), Universidade NOVA de Lisboa, Lisbon, Portugal; 9 ISPA-Instituto Universitário de Ciências Psicológicas, Sociais e da Vida, Lisbon, Portugal

**Keywords:** breast cancer, confirmatory factor analysis, PCL-5, posttraumatic growth, post-traumatic stress, psychometric validation, psycho-oncology, PTGI-SF

## Abstract

Cancer diagnosis is a stressful event that may trigger post-traumatic stress (PTS) symptoms, while also fostering positive psychological changes known as post-traumatic growth (PTG). Although distinct, PTS and PTG often co-occur among women with breast cancer. Despite their clinical relevance, validated self-report measures of PTS and PTG remain limited in oncology. This study validated the Portuguese versions of the Post-Traumatic Stress Disorder Checklist for DSM-5 (PCL-5) and the Posttraumatic Growth Inventory – Short Form (PTGI-SF) in women with breast cancer. A total of 122 women with early-stage breast cancer completed both scales at 12 and 18 months after diagnosis, considering the cancer experience as the traumatic event. Cross-sectional analyses, including confirmatory factor analyses, internal consistency estimates, and construct validity correlations, used the 12-month assessment. Test-retest reliability was examined between the 12- and 18-month assessments. Construct validity was examined with the Positive and Negative Affect Schedule – Short Form (PANAS-SF) and the European Organisation for Research and Treatment of Cancer Quality of Life Questionnaire (EORTC QLQ-C30). The PCL-5 demonstrated excellent four-factor fit, high internal consistency, and generally acceptable test-retest reliability, although reliability was poor for the Avoidance subscale. Convergent validity was supported by positive correlations with negative affect (PANAS-SF), and divergent validity by negative correlations with positive affect (PANAS-SF) and quality of life (EORTC QLQ-C30). The PTGI-SF showed a good fit to a unidimensional model, with excellent internal consistency (α = 0.91; ω = 0.91) and high test-retest reliability (ICC = 0.91), although correlations with affect and quality of life were non-significant, indicating limited convergent and divergent validities. Overall, findings support the psychometric adequacy of the PCL-5 and, to a lesser extent, the PTGI-SF, in patients with breast cancer and low PTS symptom levels. The PCL-5 appears robust for assessing PTS symptoms in this population, while the PTGI-SF captures global growth experiences, despite weaker evidence for validity. Combined use of both instruments may provide a comprehensive assessment of vulnerability and resilience following breast cancer, supporting more individualized psychosocial care and guiding future research on psychological responses to cancer, including larger and more clinically diverse samples, as patients with higher PTS symptom severity.

## 1 Introduction

Breast cancer is the most common cancer among women worldwide and represents one of the leading causes of morbidity and mortality in women (1). In 2020 there were 2.3 million new cases globally, accounting for 11.7% of all cancer diagnoses (1). In Europe, breast cancer represented 26.4% of all cancers diagnosed in women, while in Portugal 8,954 new cases were estimated in 2022, corresponding to 28.2% of all female cancer diagnoses (2). Beyond the physical impact of cancer and its treatments, the experience of the disease constitutes a highly stressful event (3).

A cancer diagnosis is increasingly conceptualized as a potential traumatic stressor, given its association with threats to survival, intrusive medical procedures, and disruption of personal, social, and occupational functioning (4). Indeed, the diagnosis of breast cancer may trigger post-traumatic stress (PTS) responses for many women, particularly due to invasive treatments, visible bodily changes, and ongoing psychosocial challenges (5). Between 20% and 30% of women report persistent psychological symptoms, including anxiety, depression, and PTS, even several years after completion of treatments (3, 6). Unlike discrete traumatic events, such as accidents, assaults, or natural disasters, which are typically time-limited and externally bounded, cancer represents a prolonged, recurrent, and internal threat (7, 8). This ongoing stressor contributes to sustained uncertainty and fluctuating perceptions of risk, which can erode coping strategies over time and increase the likelihood of long-term psychological distress (8).

Post-Traumatic Stress Disorder (PTSD) is a psychological disorder characterized by a group of symptoms that emerge following exposure to traumatic events. According to the DSM-5 (9), PTSD symptoms are grouped into four domains: (1) Intrusions (IN), which are recurrent and involuntary recollections of the event; (2) Avoidance (AV) of memories or situations associated with the trauma; (3) Negative Alterations in Cognition and Mood (NACM); and (4) Alterations in Arousal and Reactivity (AAR). Although historically associated to war veterans or victims of disasters, research has demonstrated that potentially life-threatening medical diagnoses, such as cancer, can also be sufficiently traumatic to trigger clinically relevant PTS symptoms (7, 10). The distinctive feature of cancer related trauma lies in its continuing and internal nature, in contrast to acute and external traumatic events. The body itself becomes a constant source of threat, thereby challenging emotional regulation and coping processes (8). The literature identifies multiple factors associated with the development of PTS symptoms in patients with cancer: perceived threat to life, type of treatment (surgery, chemotherapy, radiotherapy, hormone therapy), visible bodily changes, persistent pain, and subjective perceptions of control or helplessness (5, 11). Women with a prior history of trauma, lack of social support, or maladaptive coping strategies are at increased risk of post-traumatic symptomatology (11). Beyond its mental health impact, PTS symptoms are also associated with lower treatment adherence, poorer quality of life, and a higher risk of psychiatric comorbidities such as depression and anxiety (12).

A complementary perspective is offered by the concept of Posttraumatic Growth (PTG). According to Tedeschi and Calhoun (13), PTG refers to positive psychological changes that arise from the struggle with highly challenging life circumstances. It involves a meaningful reconstruction of one’s view of the self, the world, and interpersonal relationships. A substantial amount of research shows that many individuals facing life-threatening illnesses report not only post-traumatic symptoms but also positive outcomes such as renewed purpose, strengthened relationships, and greater appreciation of spirituality (14, 15). The five dimensions commonly described within PTG include: appreciation of life, enhanced interpersonal relationships, personal strength, openness to new experiences, and spiritual change (13).

Longitudinal studies suggest that PTG may develop over time, particularly when individuals have sufficient time and psychological distance to reflect upon their traumatic experience (16). Facilitating factors include perceived social support, spirituality, coping strategies, and moderate levels of stress (15, 17). Interestingly, growth does not prevent the presence of distress, rather, PTG may coexist with PTS symptoms (18). As proposed in theoretical models, emotional suffering may facilitate processes of reconstruction and personal transformation (19).

The relationship between post-traumatic stress symptoms and post-traumatic growth has been the subject of extensive research, revealing a complex and multifaceted association. Meta-analytic evidence supports the existence of both linear and curvilinear relationships between these constructs. Specifically, Shakespeare-Finch and Lurie-Beck (20) demonstrated that, while a significant linear relationship exists between PTS symptoms and PTG, a significantly stronger curvilinear relationship emerges, taking the form of an inverted-U shape. This curvilinear pattern suggests that moderate levels of PTS symptoms are associated with higher levels of growth, whereas both very low and very high levels of symptoms are linked to lower growth. However, extreme levels of stress may inhibit PTG, suggesting that some degree of distress may be necessary for growth, but excessive distress may hinder it (21).

In oncological populations, this relationship appears particularly relevant. A recent systematic review and meta-analysis conducted by Marziliano et al. (22) examined 51 studies focusing on patients and survivors of cancer, revealing a small but statistically significant positive association between post-traumatic stress and post-traumatic growth (r = 0.08). Notably, this relationship was significantly stronger among patients with advanced-stage cancer (stage 4) compared to those with non-metastatic disease, suggesting that the perceived threat to life may be a critical factor in eliciting growth experiences. On the other hand, Schubert et al. (23) emphasized that post-traumatic growth in individuals with PTSD represents a distinct psychological phenomenon, involving specific biological and psychological mechanisms that operate independently from symptom reduction. These findings underscore that PTG and PTS symptoms are not opposite ends of a continuum but rather coexisting dimensions of the trauma response, each requiring specific assessment and intervention strategies. This coexistence emphasizes the importance of assessing both domains to achieve a more comprehensive understanding of the trauma experience in oncological contexts.

Multiple psychological instruments have been developed to assess PTS symptoms and PTG. For PTS symptoms, the *Impact of Event Scale – Revised* (24) and the *Davidson Trauma Scale* (25) are widely used. However, the *PTSD Checklist for DSM-5* (PCL-5) stands out for its direct correspondence with DSM-5 diagnostic criteria, its strong construct validity, high internal consistency, and practical clinical application (26). The PCL-5 allows the assessment of both presence and severity of symptoms, supporting rapid and effective screening in clinical and research settings. This self-report scale comprises 20 items covering the four PTSD symptom clusters based on the correspondence diagnostic criteria of the DSM-5 (IN, AV, NACM, and AAR) and has excellent internal consistency (α > 0.90), strong construct validity, and sensitivity to change across trauma-exposed clinical populations (e.g., military veterans and patients exposed to medical or health-related traumatic events), as well as non-clinical community and general population samples (27, 28). In the domain of PTG, the most widely used measure is the *Posttraumatic Growth Inventory* (PTGI) (13), which has been adapted across several languages. Its short version, the *PTGI-Short Form* (PTGI-SF) (29), was developed to provide a brief and psychometrically comprehensive assessment of PTG while retaining the five original factors: Appreciation of Life (AoL), New Possibilities (NP), Personal Strength (PS), Relating to Others (RtO), and Spiritual Change (SC). Compared with other instruments such as the *Stress-Related Growth Scale* (30) or the *Benefit Finding Scale* (31), the PTGI-SF offers advantages of brevity, cross-cultural applicability, and robust empirical support (29, 32–34). It consists of 10 items reflecting five dimensions of growth and has demonstrated good reliability (α ≈ 0.85), convergent validity, and a stable factor structure, consistent with the original long version (29). In Portugal, a validated version of the PTGI-SF is available and has shown good psychometric properties in non-clinical samples, specifically among adults exposed to divorce as a potentially traumatic life event (32).

Although both instruments have been validated in community and clinical samples, including general population samples, trauma-exposed individuals, military veterans, and diverse psychosocial and medical contexts (26, 27, 29), knowledge regarding their generalizability to different cancer types remains limited. While research on post-traumatic stress and post-traumatic growth in oncology has expanded considerably since 2015 (7, 12, 14), much of this literature has focused on estimating prevalence rates and global measures of psychological adjustment, rather than on the psychometric validation of specific instruments across distinct cancer populations. This gap is particularly pronounced concerning the validation of the PCL-5 and PTGI-SF in specific cancers, such as breast cancer, which limits confidence in their clinical applicability for this population. In the case of the PCL-5, recent studies have begun to demonstrate adequate reliability and factorial validity in gastrointestinal and general cancer samples (35, 36). However, available psychometric evidence for the PCL-5 in oncology largely derives from heterogeneous cancer samples, with limited attention to disease-specific validation (35, 36). Psychometric evidence for the PTGI-SF in cancer populations is even more limited. Although post-traumatic growth has been widely studied in oncology, most research has relied on the original. Posttraumatic Growth Inventory - long version (PTGI), with fewer studies formally assessing the psychometric properties of the short form. Although the PTGI-SF was originally developed to preserve the five conceptual domains of the PTGI, evidence regarding its dimensionality across languages, cultures, and clinical contexts is not entirely uniform. The original validation supported a structure equivalent to the PTGI, with two items representing each PTG domain (29). However, validation studies in oncology and culturally diverse samples suggest that the 10-item structure may be more fragile than the long version. For example, in Malaysian cancer patients, Abdullah et al. (33) reported that a five-factor model provided the best fit, although some fit indices suggested that the model was not optimal. Similarly, work in women diagnosed with breast cancer has raised concerns regarding the adequacy of PTGI-SF versions and suggested that the dimensionality of PTG may vary depending on the population, language, and version of the instrument used (37). More recent cross-cultural psychometric evidence has also shown that shortened PTGI and PTGI-SF forms may behave as global or alternative reduced-dimensionality measures rather than fully reproducing the original five-domain structure (34). These findings suggest that, although the PTGI-SF is efficient and clinically practical, its factor structure may be sensitive to cancer type, cultural context, language profile, and sample characteristics. Validating these instruments specifically in breast cancer patients is particularly valuable given the unique psychosocial challenges associated with this disease, including body image concerns, treatment-related disruptions, and the substantial prevalence among women. In the Portuguese context, this gap is especially evident. While validated Portuguese versions of the PCL-5 (38) and the PTGI-SF (32) are available, neither has been psychometrically validated in patients with cancer.

This lack of validated tools contrasts with international psycho-oncology guidelines (39), which recommend that the systematic assessment of psychological adjustment be part of routine cancer care. In this framework, the combined use of PCL-5 and PTGI-SF is particularly useful because it allows simultaneous assessment of the traumatic impact and the potential psychological growth associated with the disease. This joint assessment of post-traumatic stress symptoms and post-traumatic growth characteristics allows clinicians to characterize each person’s psychological profile, identifying whether interventions should prioritize distress reduction, growth promotion, or both dimensions. By assessing both dimensions in an integrated manner, health professionals may implement more personalized psychosocial care, contributing to both symptom alleviation and resilience enhancement. To address this need, the main aim of the present study is to validate the Portuguese versions of PCL-5 and PTGI-SF in a sample of women with breast cancer, exploring key psychometric properties including factor structure, internal consistency, convergent validity, and test–retest reliability. By doing so, this work aims to provide reliable and valid instruments that support high-quality psychological assessment and intervention in cancer care.

## 2 Materials and methods

### 2.1 Participants

The present study was conducted at the Champalimaud Clinical Centre, within the framework of the international multicenter project BOUNCE (Predicting Effective Adaptation to Breast Cancer to Help Women to BOUNCE Back). The sample comprised 122 Portuguese women with a recent confirmed diagnosis of early breast cancer (stages I to III). Participants were recruited during their first medical appointment with an oncologist, prior to the initiation of any systemic treatment. After enrolment in the study, participants completed the baseline assessment, followed by periodic reassessments in accordance with the study protocol (40).

Inclusion criteria required participants to be female, aged between 18 and 70 years at the time of diagnosis, to have a first diagnosis of breast cancer (stages I to III), and to be eligible for systemic treatment. Exclusion criteria included the presence of metastatic disease, previous history of neoplasia (except for successfully treated basal cell carcinoma of the skin or *in situ* carcinoma of the cervix), previous major psychiatric diagnoses such as schizophrenia, psychosis, bipolar disorder, or major depression, as well as neurological disorders, clinically significant cardiac disease, or recent major surgery with potential impact on psychosocial well-being (40).

Sociodemographic information was collected through a self-report questionnaire developed specifically for the BOUNCE study, including information on age, marital status, educational level and employment status. Clinical data was obtained through the review of medical records and included information on tumor stage, type of treatment, and the presence of comorbidities. Participants were also invited to complete a battery of questionnaires designed to assess their quality of life, emotional state, and other relevant psychosocial variables.

### 2.2 Instruments

#### 2.2.1 PTSD checklist for DSM-5

The PCL-5 (26, 38, 41) is a 20-item self-report scale that assesses the presence and severity of PTS symptoms based on the PTSD diagnostic criteria of the DSM-5 and it was not used to establish a clinical diagnosis of PTSD. The items are grouped into four domains: Intrusions (IN), Avoidance (AV), Negative Alterations in Cognition and Mood (NACM), and Alterations in Arousal and Reactivity (AAR). The participants rated how much they had been bothered by each symptom over the past month, in relation to their breast cancer experience. Responses are given on a 5-point Likert scale, ranging from 0 (“Not at all”) to 4 (“Extremely”). The total score is obtained by adding all 20 items, with higher scores representing greater post-traumatic symptoms. The PCL-5 was used as a self-report measure of PTS symptom severity and was not used to establish a clinical diagnosis of PTSD. In the original validation study, the PCL-5 demonstrated excellent internal consistency (total α = 0.94; IN α = 0.87; AV α = 0.81; NACM α = 0.87; AAR α = 0.90), strong test–retest reliability (r = 0.82), and convergent validity with measures of depression, anxiety, and stress (r ≈ 0.70) (26). The Portuguese version also showed strong psychometric properties in community and clinical samples exposed to heterogeneous traumatic events (non-oncological trauma-exposed samples), including excellent internal consistency (total α = 0.94; IN α = 0.89; AV α = 0.76; NACM α = 0.87; AAR α = 0.85), high test–retest reliability over a three-week interval (IN r = 0.91; AV r = 0.88; NACM r = 0.90; AAR r = 0.89), and convergent validity through significant correlations with depression, anxiety, and stress symptom severity (r = 0.39–0.63) (38). Importantly, in both validation studies, PTSD symptoms were assessed in relation to heterogeneous post-traumatic experiences, without anchoring symptom assessment to a specific medical diagnosis or treatment-related timepoint (26, 38).

#### 2.2.2 Posttraumatic growth inventory – short form

The PTGI-SF (29, 32) comprises 10 items that assess perceived positive changes following a potentially traumatic event. The items are distributed across five dimensions: Appreciation of Life (AoL), New Possibilities (NP), Personal Strength (PS), Relating to Others (RtO) and Spiritual Change (SC). Responses are provided on a 6-point Likert scale, ranging from 0 (“I did not experience this change”) to 5 (“I experienced this change to a very great degree”). For this scale, responses were anchored to perceive positive changes resulting from the breast cancer experience, without a fixed recent time window. The total score is obtained by adding all items, with higher scores indicating greater post-traumatic growth. In the original study, internal consistency was high (α = 0.90) (29), and the Portuguese version similarly showed good reliability (α = 0.86) (32). Additionally, the Portuguese validation study demonstrated satisfactory convergent validity through a moderate correlation with the Growth Motivation Index (r = 0.44, p < 0.001), supporting the association between post-traumatic growth and personal motivation for self-improvement and psychological growth (32). In both validation studies, post-traumatic growth was assessed in relation to non-medical life events or heterogeneous past stressors, with participants instructed to consider a personally meaningful stressful or traumatic event when responding to the items (29, 32). In the present study, posttraumatic growth was explicitly assessed in relation to the experience of breast cancer.

#### 2.2.3 European organization for research and treatment of cancer – quality of life questionnaire - Core 30

The EORTC QLQ-C30 (42, 43) is a 30-item questionnaire designed to assess quality of life in individuals with oncological diseases. It comprises functional, symptom, and global quality of life subscales. In the present study, only the Global Quality of Life subscale was used, consisting of two items (items 29 and 30) rated on a 7-point Likert scale ranging from 1 (“Very poor”) to 7 (“Excellent”) with reference to the past week. The internal consistency of the global subscale of the Portuguese version was 0.86 (43). The EORTC QLQ-C30 was included in this study to assess divergent validity of the PCL-5 and its four symptom clusters, and convergent validity of the PTGI-SF, based on theoretical models distinguishing post-traumatic stress, post-traumatic growth, and quality of life (17, 44, 45).

#### 2.2.4 Positive and negative affect schedule – short form

The PANAS-SF (46, 47) is a 10-item self-report measure assessing affective states over the past week. It includes five items for Positive Affect (enthusiastic, inspired, alert, active, determined) and five items for Negative Affect (afraid, distressed, nervous, upset, guilty). Responses are rated on a 5-point Likert scale ranging from 1 (“Very slightly or not at all”) to 5 (“Extremely”). The total score is calculated by summing the items for each subscale: higher scores on the Positive Affect subscale reflect greater positive emotional activation, whereas higher scores on the Negative Affect subscale reflect greater negative emotional activation. The Portuguese version demonstrated good internal consistency in the original validation study (Positive Affect α = 0.81; Negative Affect α = 0.82) (46). In the present study, PANAS-SF was included to assess construct validity. For the PCL-5, the Negative Affect subscale was included to assess convergent validity of the total score and symptom clusters, and the Positive Affect subscale was included to assess divergent validity. For the PTGI-SF, the Positive Affect subscale was included to assess convergent validity, whereas the Negative Affect subscale was included to assess divergent validity, in line with theoretical models distinguishing post-traumatic stress, post-traumatic growth, and affective functioning (17, 44, 47).

### 2.3 Procedures

This study was conducted within the framework of the BOUNCE project, funded by the European Union’s Horizon 2020 research and innovation program under Grant Agreement No. 777167. The study protocol was approved by the European Institute of Oncology Ethics Committee (approval No. R868/18-IEO 916; approval date: 24 October, 2018) as the coordinating center, and subsequently by the Ethics Committee of the Champalimaud Clinical Centre, and all procedures were carried out in accordance with the ethical principles of the Declaration of Helsinki. Participants were fully informed about the study objectives and provided written informed consent prior to data collection.

All data processing was conducted in compliance with ethical standards for scientific research and with international, European, and national legislation, particularly the General Data Protection Regulation (GDPR, EU 2016/679). Data access was restricted to authorized members of the research team, ensuring confidentiality, anonymity, and participant safety throughout all stages of the study. Previously validated Portuguese versions of all instruments were used in this study. No additional translation or cultural adaptation procedures were required, as validated versions of all scales were already available (32, 38, 43, 46).

### 2.4 Statistical analyses

Data analyses were performed using IBM SPSS Statistics version 30; AMOS version 30; and JASP version 18.0, based on the lavaan package for R.

Descriptive statistics were calculated for the sociodemographic and psychometric variables, including means (M) and standard deviations (SD), minimum and maximum values, percentages of endorsement, skewness (Sk), and kurtosis (Ku).

All cross-sectional psychometric analyses were conducted using data from the 12-month assessment. Specifically, item-level descriptive statistics, confirmatory factor analyses (CFA), internal consistency estimates, and construct validity correlations were based on 12-month data. This assessment was selected as the primary psychometric timepoint because both instruments were available at this moment and because it represents a clinically meaningful post-diagnosis interval, during which initial treatment-related adaptation processes may have begun to stabilize while still remaining sufficiently close to the cancer experience under evaluation.

No *a priori* hypotheses were established regarding baseline skewness, floor effects, or restricted symptom variability for either the PCL-5 or the PTGI-SF. Instead, we adopted a descriptive empirical characterization of item distributions, including response endorsement patterns, skewness, and kurtosis. These analyses were used to describe the psychometric behavior of the items in this specific oncology sample and to inform the interpretation of subsequent results. This descriptive examination was not an exploratory factor analytic approach. Because the present study followed a confirmatory psychometric approach, the factorial analyses were specified *a priori* based on the original psychometric models of the instruments, their Portuguese adaptations, and available international psychometric evidence, with particular attention to oncology contexts.

To assess dimensionality of the PCL-5 and PTGI-SF scales, CFA were conducted based on the theoretical models proposed by the authors of the respective original scales. For the PCL-5, the four-factor model based on the DSM-5 was considered (26). For the PTGI-SF, Cann et al. (29) originally evaluated three competing CFA models: a unidimensional model, a five correlated factors model, and a hierarchical model with five first-order factors and one second-order general factor. However, in our study, only the unidimensional model was specified as the primary solution, given the sample size and the methodological constraints associated with estimating models composed of multiple two-item factors (48, 49). Parameter estimation was performed using the Diagonally Weighted Least Squares (DWLS) method, which is recommended for ordinal response scales and moderate sample sizes. Model fit was assessed using multiple indices, including the chi-square statistic (χ²), with non-significant values (p > 0.05) indicating good fit; the ratio of chi-square to degrees of freedom (χ²/df), with values ≤ 3.0 indicating acceptable fit; the Comparative Fit Index (CFI) and Tucker–Lewis Index (TLI), for which values ≥ 0.90 indicate good fit and values ≥ 0.95 indicate excellent fit; and the Root Mean Square Error of Approximation (RMSEA) and the Standardized Root Mean Square Residual (SRMR), with values ≤ 0.08 indicating acceptable fit and values ≤ 0.05 indicating very good fit (50, 51).

Item quality was examined through factor loadings, with loadings ≥ 0.40 considered adequate, reflecting a strong association between the latent and observed variables. Scale reliability was assessed using Cronbach’s alpha (α) and McDonald’s omega (ω) coefficients, with values ≥0.70 regarded as indicative of good internal consistency (51). Additionally, corrected item–total correlations were analyzed, with values ≥ 0.30 deemed acceptable for item homogeneity (52). Temporal stability was evaluated using intraclass correlation coefficients (ICC), comparing the scores obtained 12 and 18-months after the baseline assessment. For all analyses, a significance level of *p* < 0.05 was adopted.

In addition to test–retest reliability analyses, PCL-5 and PTGI-SF total scores were compared between the 12- and 18-month assessments to provide a descriptive evaluation of change across the two repeated-measurement timepoints. The normality of difference scores was examined to determine the appropriate paired-sample test. Because PCL-5 difference scores departed from normality, the Wilcoxon signed-rank test was used for this comparison. Because PTGI-SF difference scores did not significantly deviate from normality, a paired-samples t-test was used for this outcome. Because not all participants completed both instruments at the 18-month assessment, repeated-measures analyses were conducted using the available paired data for each scale. Accordingly, sample sizes differ across the 12–18-month comparisons, reflecting scale-specific availability of complete paired observations rather than attrition from the primary 12-month psychometric sample.

To assess construct validity, separate Pearson correlations were calculated for each scale. For the PCL-5, convergent validity was assessed through correlations with PANAS-SF Negative Affect, while divergent validity was evaluated against the EORTC QLQ-C30 and PANAS-SF Positive Affect. For the PTGI-SF, convergent validity was examined in relation to the EORTC QLQ-C30 and PANAS-SF Positive Affect, whereas divergent validity was evaluated through correlations with PANAS-SF Negative Affect.

## 3 Results

### 3.1 Descriptive statistics

The sample consisted of 122 Portuguese women diagnosed with breast cancer, aged between 40 and 70 years (*M* = 53.22, *SD* = 8.10). Regarding clinical characteristics, all participants had early-stage, non-metastatic breast cancer, with most participants diagnosed at stage I (45.1%) or II (45.1%). A full description of sociodemographic and clinical characteristics is provided in **Table 1**.

**Table 1:** Sociodemographic characteristics of the sample.

Sociodemographic and clinical characteristics (N = 122)	*n*	*%*
Age. M (SD)	53.22 (8.10) [Min. (40); Max. (70)]	
Age group		
≤40 years	2	1.6
41–50 years	52	42.6
51–60 years	42	34.4
>60 years	26	21.3
Highest level of education		
Primary	4	3.3
Lower secondary	7	5.7
Higher secondary	18	14.8
Post-secondary non-graduate	65	53.2
Graduate degree	28	23.0
Marital status		
Single/Engaged	12	9.8
Married	94	77.0
Divorced/Widowed	16	13.2
Employment status		
Employed	94	77.0
Unemployed/Housewife	10	8.2
Retired	18	14.8
Cancer stage at diagnosis		
Stage I	55	45.1
Stage II	55	45.1
Stage III	12	9.8

N, total sample size; n, number of participants in each category; M, mean; SD, standard deviation; Min., minimum; Max., maximum.

Descriptive statistics for the individual items of each scale are presented in **Table 2** (PCL-5) and **Table 3** (PTGI-SF). For the PCL-5, the percentage of responses for each item shows a clear tendency toward lower ratings, with most participants selecting the two lowest response categories (0 “Not at all” and 1 “A little bit”) in more than 60% of all items, indicating that most symptoms were reported as absent or only mildly present. Consistently, kurtosis values greater than 3 were observed for items 9, 10, 11 and 14, suggesting highly peaked distributions, with these and items 2, 3, 5 and 16 displaying pronounced positive skewness (Sk > 1.5), indicating strong clustering of responses at the lower end of the scale (**Table 2**). Overall, these results show non-normal item distributions, which is common in PTS symptoms self-report data and support the use of robust estimators suitable for ordinal data in subsequent factor analyses, such as DWLS (53, 54).

**Table 2:** Individual PCL-5 item summaries for the total sample at the 12-month assessment.

Item	Statistics			Percentage of endorsement					
	M(SD)	Sk	Ku	0	1	2	3	4	Total
1	1.04 (0.94)	0.57	-0.55	32.8	39.1	19.5	8.6	0.0	100
2	0.49 (0.79)	1.56	1.65	66.4	21.1	9.4	3.1	0.0	100
3	0.41 (0.73)	1.81	2.76	70.3	20.3	7.0	2.3	0.0	100
4	0.80 (0.87)	0.98	0.70	43.8	36.7	15.6	3.1	0.8	100
5	0.59 (0.88)	1.55	1.59	60.9	26.6	5.5	7.0	0.0	100
6	1.08 (1.15)	0.88	-0.28	39.8	32.0	11.7	13.3	3.1	100
7	0.91 (1.08)	1.11	0.39	46.5	30.7	11.0	9.4	2.4	100
8	0.61 (0.88)	1.21	0.30	61.7	19.5	14.8	3.9	0.0	100
9	0.34 (0.72)	2.43	5.58	77.3	15.6	3.1	3.9	0.0	100
10	0.49 (0.87)	1.92	3.24	68.8	19.5	6.3	4.7	0.8	100
11	0.45 (0.73)	2.05	5.27	65.6	27.3	4.7	1.6	0.8	100
12	0.60 (0.80)	1.41	1.75	54.7	35.2	5.5	4.7	0.0	100
13	0.66 (0.81)	1.16	0.83	51.6	34.9	9.5	4.0	0.0	100
14	0.32 (0.65)	2.69	9.47	75.0	20.3	3.1	0.8	0.8	100
15	0.55 (0.72)	1.32	1.63	56.3	35.2	6.3	2.3	0.0	100
16	0.31 (0.55)	1.53	1.45	72.4	23.6	3.9	0.0	0.0	100
17	1.02 (1.05)	0.82	-0.19	39.1	32.8	16.4	10.2	1.6	100
18	0.77 (0.76)	0.86	0.62	39.8	46.9	10.2	3.1	0.0	100
19	1.12 (0.99)	0.82	0.16	28.1	45.3	14.8	10.2	1.6	100
20	1.35 (1.14)	0.54	-0.73	25.8	37.5	15.6	18.0	3.1	100

PCL-5, PTSD Checklist for DSM-5; M, mean; SD, standard deviation; Sk, skewness; Ku, kurtosis; Items are presented according to their order in the PCL-5; Values under “Percentage of endorsement” represent the proportion (%) of participants selecting each response category (0–4) on the PCL-5.

For the PTGI-SF, in contrast, item responses showed a more balanced and normalized distribution across lower scores (response options 0–1), intermediate scores (2–3), and higher scores (4–5). Skewness values ranged from –0.85 to 0.61 and kurtosis values from –1.32 to –0.39, indicating no relevant violations of univariate normality (**Table 3**). These distributional properties support the suitability of conducting CFA using Diagonally Weighted Least Squares (DWLS) for ordinal data.

**Table 3:** Individual PTGI-SF item summaries for the total sample at the 12-month assessment.

Item	Statistics				Percentage of endorsement					
	M(SD)	Sk	Ku	0	1	2	3	4	5	Total
1	2.55 (1.47)	-0.26	-0.89	15.3	11.5	10.5	32.3	27.4	3.2	100
2	3.05 (1.53)	-0.76	-0.45	11.3	8.1	8.1	23.4	35.5	13.7	100
3	2.60 (1.45)	-0.46	-0.87	12.1	14.5	10.5	31.5	26.6	4.8	100
4	1.96 (1.74)	0.29	-1.32	31.5	16.1	10.5	17.7	15.3	8.9	100
5	2.46 (1.56)	-0.33	-1.04	19.5	8.9	13.0	30.1	22.0	6.5	100
6	2.08 (1.60)	0.00	-1.30	26.6	11.3	16.9	21.8	19.4	4.0	100
7	2.96 (1.52)	-0.63	-0.62	10.5	10.5	8.9	25.8	31.5	12.9	100
8	1.60 (1.67)	0.61	-0.93	41.1	13.7	12.1	17.7	8.1	7.3	100
9	3.26 (1.57)	-0.85	-0.39	9.7	8.9	6.5	17.7	35.5	21.8	100
10	2.70 (1.66)	-0.41	-1.04	17.7	8.9	9.7	25.8	25.0	12.9	100

PTGI-SF, Posttraumatic Growth Inventory–Short Form; M, mean; SD, standard deviation; Sk, skewness; Ku, kurtosis; Items are presented according to their order in the PTGI-SF; Values under “Percentage of endorsement” represent the proportion (%) of participants selecting each response category (0–5) on the PTGI-SF.

### 3.2 Dimensionality

CFA was conducted using data from the 12-month assessment to analyze the factorial structure of both the PCL-5 and the PTGI-SF, using the DWLS estimation method. Although the distributional properties differed between instruments, both scales comprise ordinal Likert-type items and the DWLS estimator adopted for the CFA’s is robust to non-normality and specifically recommended for ordinal data, particularly when item distributions deviate from normality, as observed for the PCL-5 (53, 54). For the PCL-5, the four-factor model (IN, AV, NACM, and AAR) demonstrated excellent fit (χ² = 112.26, df = 164, χ²/df = 0.68, p > 0.999; CFI = 1.00; TLI = 1.03; RMSEA = 0.00), with SRMR = 0.08 suggesting acceptable fit. All factor loadings were statistically significant (p < 0.001), ranging from 0.45 to 0.89, indicating moderate to strong associations between the items and their respective latent constructs (**Figure 1**; **Table 4**).

**Figure 1:**
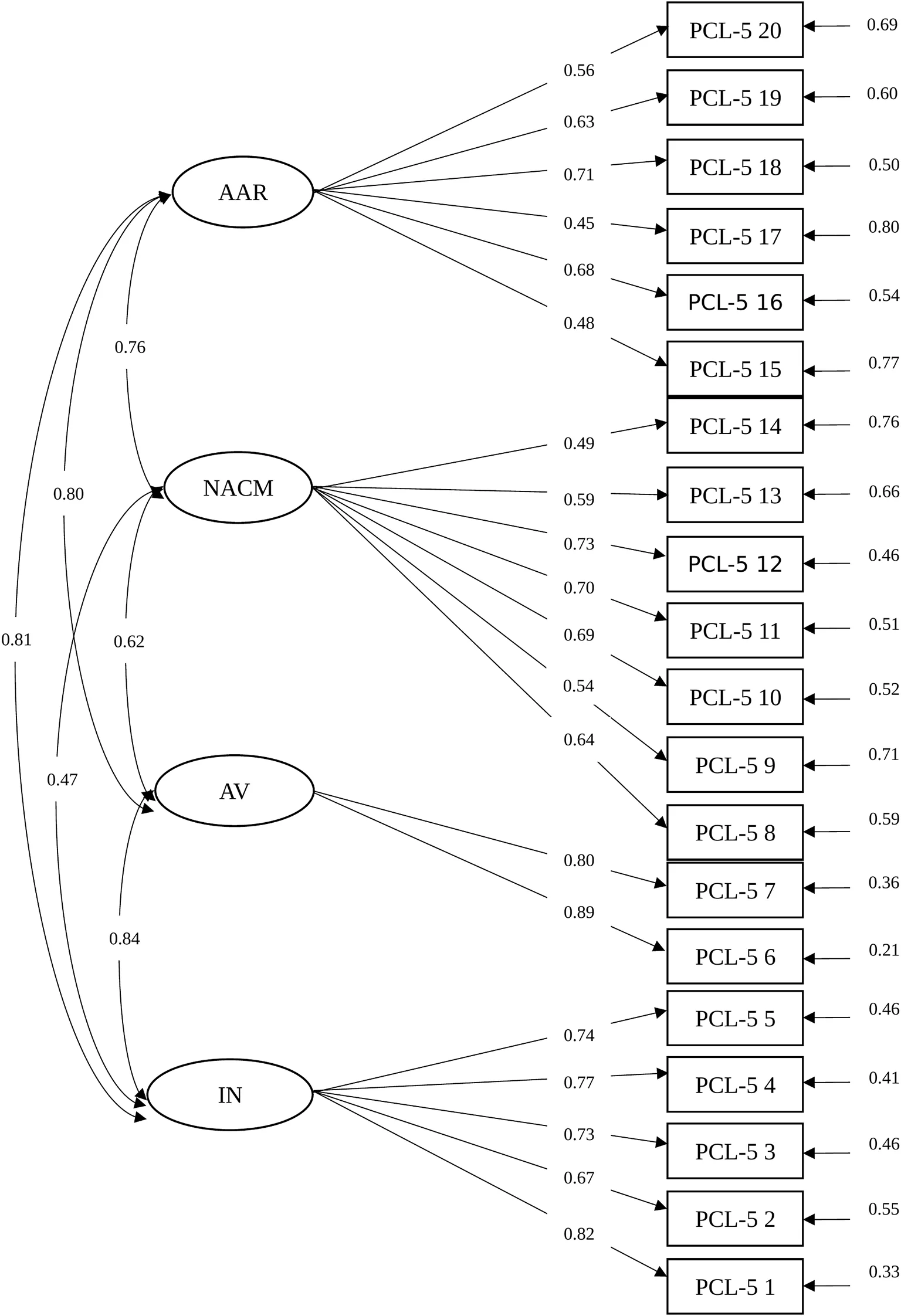
Confirmatory factor analysis of the four-factor model of the PCL-5. Standardized factor loadings and measurement error variances are displayed: Inter-factor correlations are presented on the left side of the diagram (curved arrows between latent factors), standardized factor loadings on the paths linking factors to items (center), and measurement error variances on the right of each item.

**Table 4:** CFA model fit indices for PCL-5 and PTGI-SF.

Scale	Model	χ²	*df*	χ²/df	CFI	TLI	RMSEA (90% CI)	SRMR
PCL-5	Four-factor DSM-5 model	112.26	*164*	0.68	1.00	1.03	0.00 [0.00-0.00]	0.08
PTGI-SF	Unidimensional model	55.76	35	1.59	0.99	0.98	0.07 [0.03-0.10]	0.09

CFA, confirmatory factor analysis; PCL-5, PTSD Checklist for DSM-5; PTGI-SF, Posttraumatic Growth Inventory–Short Form; χ², chi-square statistic; df, degrees of freedom; CFI, Comparative Fit Index; TLI, Tucker–Lewis Index; RMSEA, Root Mean Square Error of Approximation; SRMR, Standardized Root Mean Square Residual.

Regarding the PTGI-SF, the unidimensional model was examined as the primary solution. The unidimensional model showed an overall adequate fit (χ² = 55.76, *df* = 35, χ²/df = 1.59, *p* = 0.014; CFI = 0.99; TLI = 0.98; RMSEA = 0.07; SRMR = 0.09) with all factor loadings statistically significant (*p* < 0.001), ranging from 0.60 to 0.83. These results support the adequacy of the unidimensional structure in the present sample, with all PTGI-SF items displaying moderate to strong standardized factor loadings on a single latent factor (λ = 0.60–0.83; **Figure 2**; **Table 4**). This is consistent with psychometric evidence supporting a strong general post-traumatic growth factor underlying the PTGI-SF, as reflected in the unidimensional solution reported by Fekih-Romdhane et al. (34).

**Figure 2:**
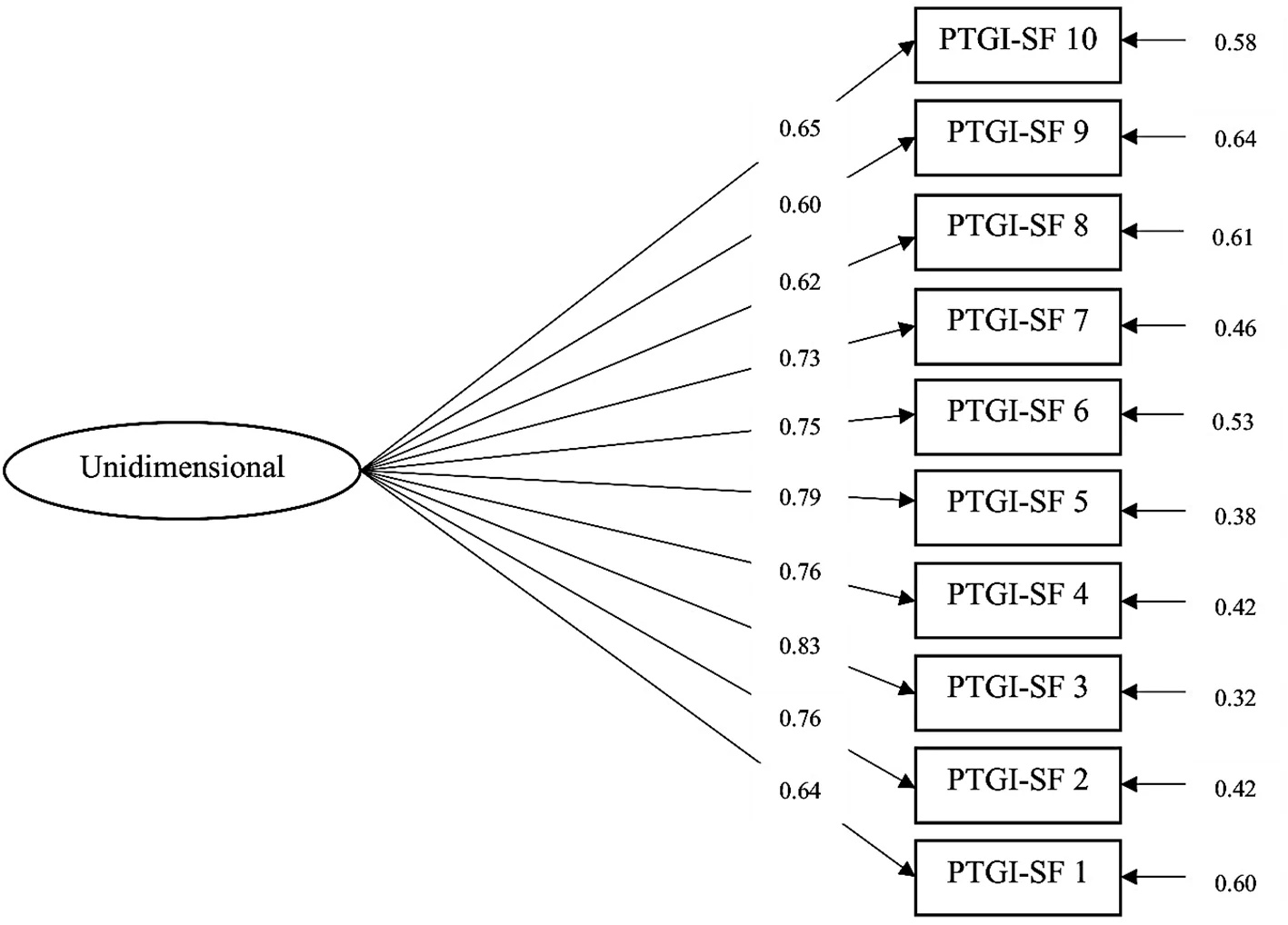
Confirmatory factor analysis of the unidimensional model of the PTGI-SF. Standardized factor loadings are displayed on the paths linking the latent factor to the items, and measurement error variances on the right of each item.

### 3.3 Internal consistency and test-retest reliability

The PCL-5 demonstrated very good internal consistency across all factors (IN: α = 0.86, ω = 0.86; AV: α = 0.83, ω = 0.84; NACM: α = 0.85, ω = 0.75; AAR: α = 0.74, ω = 0.72). Item–total correlations were all above 0.30, and no item removal produced a meaningful improvement in internal consistency (Cronbach’s α if item deleted: 0.83–0.86; McDonald’s ω if item deleted: 0.75–0.86), indicating that all items contributed adequately to their respective subscales. Test–retest reliability between the 12- and 18-month assessments was evaluated using intraclass correlation coefficients (ICC), based on a two-way mixed effects model with absolute agreement. ICC values ranged from 0.37 to 0.69 across factors (IN = 0.55, 95% CI [0.39, 0.67]; AV = 0.37, 95% CI [0.19, 0.53]; NACM = 0.65, 95% CI [0.52, 0.75]; AAR = 0.69, 95% CI [0.57, 0.78]; all p < 0.001).

The PTGI-SF showed excellent internal consistency (α = 0.91; ω = 0.91). Item–total correlations were all above 0.30, and neither Cronbach’s alpha or McDonald’s omega increased when any item was removed (α if item deleted = 0.89–0.91; ω if item deleted = 0.90–0.91), indicating that all items contributed meaningfully to the total scale and supporting the adequacy of the unidimensional structure. Test–retest reliability calculated using a two-way mixed effects model with absolute agreement was high (ICC = 0.91). The 95% confidence interval indicated excellent temporal stability over the 6-month interval (ICC = 0.91, 95% CI [0.86, 0.94], p < 0.001).

### 3.4 Comparison of PCL-5 and PTGI-SF scores between 12 and 18 months

To improve transparency regarding the repeated-measures component of the study, PCL-5 and PTGI-SF total scores were compared between the 12- and 18-month assessments. Analyses were based on participants with complete paired data for each scale, resulting in n = 93 for the PCL-5 comparison and n = 102 for the PTGI-SF comparison. Descriptive statistics and effect sizes are presented in **Table 5**. Because PCL-5 difference scores departed from normality, changes in PCL-5 total scores were examined using the Wilcoxon signed-rank test. No significant difference was observed between 12 months (M = 13.45, SD = 10.54) and 18 months (M = 13.40, SD = 11.33), Z = −0.79, p = .432, r = .08. For the PTGI-SF, difference scores did not significantly depart from normality, and a paired-samples t-test was therefore used. PTGI-SF total scores did not differ significantly between the 12-month assessment (M = 23.99, SD = 12.14) and the 18-month assessment (M = 23.03, SD = 12.72), t(101) = 1.34, p = .184, d = .13. These effect sizes were small, supporting the interpretation of relative stability across the six-month interval.

**Table 5:** Comparison of PCL-5 and PTGI-SF total scores between 12 and 18 months.

Scale	N	12-month assessment M(SD)	18-month assessment M(SD)	Test statistic	*p*	Effect size
PCL-5 total score	93	13.45 (10.54)	13.40 (11.33)	Z = -0.79	0.432	r = 0.08
PTGI-SF total score	102	23.99 (12.14)	23.03 (12.72)	T(101) = 1.34	0.184	d = 0.13

PCL-5, PTSD Checklist for DSM-5; PTGI-SF, Posttraumatic Growth Inventory–Short Form; M, mean; SD, standard deviation; PCL-5 scores were compared using the Wilcoxon signed-rank test because the distribution of difference scores departed from normality; the effect size is reported as r. PTGI-SF scores were compared using a paired-samples t-test because difference scores did not significantly deviate from normality; the effect size is reported as Cohen’s d. Analyses were based on participants with complete paired data for each scale.

### 3.5 Convergent and divergent validities

For the PCL-5 total score, convergent validity was confirmed through a significant positive correlation with PANAS-SF Negative Affect (r = 0.67, *p* < 0.001). Divergent validity was supported by significant negative correlations with EORTC QLQ-C30 (r = −0.51, p < 0.001) and PANAS-SF Positive Affect (r = −0.43, *p* < 0.001). Regarding the four PCL-5 symptom clusters, convergent validity was further evidenced by significant positive correlations with negative affect, ranging from r = 0.42 to r = 0.63 (all *p* < 0.001). Divergent validity was supported by negative correlations with positive affect (r = −0.24 to r = −0.50), and with EORTC QLQ-C30 (r = −0.25 to r = −0.50; all *p* < 0.01; **Table 6**).

**Table 6:** Convergent and divergent validities of the PCL-5.

External measure	PCL total	IN	AV	NACM	AAR
Convergent validity					
PANAS-SF Negative	0.67***	0.58***	0.42***	0.59***	0.63***
Divergent validity					
EORTC QLQ-C30 QoL	-0.51***	-0.42**	-0.25**	-0.48***	-0.50***
PANAS-SF Positive	-0.43***	-0.28**	-0.24**	-0.50***	-0.40***

***p < 0.001, **p < 0.01.

Analysis of convergent validity for the total PTGI-SF revealed non-significant correlations with the EORTC QLQ-C30 (*r* = 0.06, *p* = 0.53) and with PANAS-SF Positive Affect (*r* = 0.08, *p* = 0.38), suggesting a weak or non-existent association between these constructs. Regarding divergent validity, correlation with PANAS-SF Negative Affect was also non-significant (*r* = –0.01, *p* = 0.95).

## 4 Discussion

The present study aimed to examine the psychometric properties of the PCL-5 and the PTGI-SF in a sample of Portuguese women diagnosed with early breast cancer. Through a comprehensive assessment of factorial structure, reliability (including internal consistency and temporal stability), and construct validity, the results provide evidence supporting the adequacy of both instruments for research and clinical use in psycho-oncology. Importantly, this study contributes with novel data to validate measures of trauma-related and post-traumatic growth in oncology.

The use of overlapping criterion measures (PANAS-SF and EORTC QLQ-C30) to validate both instruments requires methodological clarification. Rather than indicating redundancy, this approach was strategically designed to test the discriminant validity of post-traumatic stress and post-traumatic growth as distinct psychological constructs. By hypothesizing differential patterns of association with the same external criteria, we sought to demonstrate that PCL-5 and PTGI-SF capture unique variance in psychological functioning. This coincidence of scales at the same assessment timepoint reflects the design of the BOUNCE project, and aligns with recommendations for construct validation, wherein demonstrating differential relationships with common criteria strengthens evidence for discriminant validity (55).

The PCL-5 showed an excellent fit to the four-factor DSM-5 model, replicating the structure originally proposed by Blevins et al. (26) and consistently observed in different contexts (27, 28). The strength of factor loadings and the internal consistency of each subscale further support the reliability of the factorial structure in a sample of patients with cancer. Although the Tucker–Lewis Index (TLI) slightly exceeded the conventional upper bound of 1.00, this phenomenon has been documented in confirmatory factor analyses of models exhibiting very high fit, particularly when incremental indices are computed relative to a poorly fitting baseline model (56, 57). Indeed, inspection of the baseline model (null model) revealed an extremely poor fit (χ²/df = 12.42), which is common in clinical scales with strongly correlated items. This pattern is further accentuated when robust estimators such as diagonally weighted least squares (DWLS) are applied to ordinal and highly skewed items, as this approach tends to yield substantially lower chi-square values and minimal residuals under correctly specified models (53, 54, 58). In contrast, the proposed four-factor model showed a substantially reduced χ²/df ratio (0.68), indicating a marked improvement in fit relative to the baseline model (56). Given that the TLI is an incremental fit index reflecting relative improvement over the null model, this large contrast explains the observed TLI value exceeding 1.00 and supports its interpretation as an indicator of model robustness rather than overfitting or misspecification (56, 57). In the present study, we focus on self-reported PTS symptom severity as assessed by the PCL-5, rather than on a clinical diagnosis of PTSD.

Notably, even though the Avoidance factor consists of only two items, a situation that can pose statistical challenges in CFA due to reduced degrees of freedom, the robust factor loadings, adequate residuals, and recommended fit indices indicate that the model is stable and conceptually coherent. This finding aligns with methodological literature in which two-item latent factors may yield acceptable results and reliable estimations when the sample size is adequate, indicators are strongly correlated, and a robust estimator such as DWLS is applied (49, 56, 57). The successful replication of the DSM-5 model in a Portuguese breast cancer sample represents a significant contribution to the literature. This specific validation is particularly valuable given that breast cancer presents unique psychosocial challenges, such as body image concerns, treatment-related interruptions, and substantial prevalence among women, which may differentially influence trauma symptom expression compared to other cancer types. Confirmation of the PCL-5’s psychometric adequacy in this specific population reinforces not only the cross-cultural generalizability of the instrument but also its clinical relevance for assessing persistent trauma-related symptoms arising from ongoing medical procedures and uncertainty characteristic of the breast cancer experience (7, 8, 27).

Construct validity analyses provided further support for the psychometric adequacy of the PCL-5. As expected, and consistently with previous research (26, 45, 47), PTS symptoms were positively correlated with negative affect and negatively correlated with both positive affect and quality of life. These patterns reinforce the convergent and discriminant validity of the PCL-5 in relation to clinically relevant indicators of emotional distress in patients with cancer. The consistency of these findings with earlier psycho-oncology studies suggests that the PCL-5 adequately captures the nuanced psychological burden associated with breast cancer diagnosis and treatment, which includes intrusive thoughts, somatic hyperarousal and persistent emotional dysregulation (7, 11). Temporal stability analyses further supported the reliability of the PCL-5. In line with these findings, the additional comparison of total scores showed no significant change in PCL-5 scores between the 12- and 18-month assessments. However, this stability should be interpreted cautiously. Although Cordova et al. (11) suggested that trauma-related symptoms may persist after breast cancer treatment, that study was not longitudinal and was based on a relatively small sample. Broader person-centered literature indicates that PTS responses after cancer-related experiences may follow heterogeneous trajectories, including stable-low or resilient, recovery, chronic, and worsening patterns (59). Accordingly, the stability observed in the present study should be interpreted as specific to this relatively low-symptom, early-stage breast cancer cohort, rather than as evidence of a uniform trajectory of PTS symptoms across oncology populations. Although the PCL-5 showed acceptable temporal stability overall, the AV factor presented the lowest test–retest reliability coefficient (ICC = 0.37). According to commonly used ICC benchmarks, this value falls within the poor reliability range (60). This finding indicates that the Avoidance subscale should not be relied upon for longitudinal monitoring or clinical decision-making in isolation. The lower stability of this factor may partly reflect its two-item composition, a known methodological challenge in latent variable models, and the possibility that AV symptoms fluctuate over time as patients move through different stages of treatment and adaptation (11, 26, 56, 57). Therefore, interpretation of the AV subscale should be made with caution, and the PCL-5 total score and broader symptom-cluster profile may provide more stable information for longitudinal assessment in this population.

For the PTGI-SF, the factorial assessment revealed that the unidimensional model provided an adequate and methodologically appropriate representation of posttraumatic growth in this sample. Although the scale was originally conceptualized to reflect five theoretically distinct factors, namely Appreciation of Life (AoL), New Possibilities (NP), Personal Strength (PS), Relating to Others (RtO) and Spiritual Change (SC) (29), our sample size limited the stable estimation of models composed of several two-item factors. This limitation is consistent with methodological constraints associated with models in which all latent factors are specified by only two indicators, which often require substantially larger sample sizes (N > 400) to achieve stable estimations (48, 61). This issue differs from situations where a single two-item factor is embedded within a well-identified multidimensional model (56, 57), as observed for the Avoidance factor of the PCL-5 scale. Therefore, in the present sample, the unidimensional model demonstrated adequate fit indices and strong internal consistency.

This pattern is compatible with the study by Fekih-Romdhane et al. (34), supporting that the short form of the PTGI often behaves as a global measure rather than a multidimensional instrument. This differs from that reported by Cann et al. (29), whose study indicated that the hierarchical model, comprising five specific factors and one general factor, showed the best fit. The emergence of a general factor in the present study may reflect several contributing factors. First, the specific nature of our breast cancer sample may have fostered a more integrated perception of growth. Unlike the heterogeneous samples employed by Cann et al. (29), which encompassed diverse traumatic events including bereavement, intimate partner violence, and medical trauma across various conditions, our study focused exclusively on women with breast cancer. This homogeneity may have facilitated a unified narrative of transformation, as the specific challenges of breast cancer, such as body image concerns, treatment-related interruptions, and ongoing uncertainty, may promote a common rather than a compartmentalized experience of growth. Second, cultural factors may have contributed to this unidimensional structure. Portuguese women facing breast cancer may interpret and integrate growth experiences differently from participants in other cultural contexts, potentially emphasizing collective or global perceptions of change over distinct, separable domains. Third, the emergence of a general factor may also reflect the phenomenology of post-traumatic growth in oncological populations more broadly, where growth experiences may be integrated, rather than separated into distinct domains (11, 44). This pattern is further supported by psychometric evidence showing that PTGI-SF total scores relate coherently to core emotion regulation processes, consistent with a global and integrated construct of post-traumatic growth (34). From a theoretical perspective, these findings align with models conceptualizing PTG as a broad restructuring of core beliefs, particularly regarding self-perception, interpersonal relationships, and life philosophy/purpose in life (17).

In contrast to PCL-5, the PTGI-SF demonstrated limited convergent and divergent validity, with correlations with positive affect, negative affect, and quality of life failing to reach statistical significance. Although these findings may suggest that perceived PTG is relatively independent from affective functioning and global quality of life, they should be interpreted cautiously. The absence of significant associations may also reflect limited statistical power, restricted variability in this relatively homogeneous sample of women with early-stage breast cancer, measurement error associated with the 10-item short form, or limited sensitivity of a unidimensional PTGI-SF solution to capture more nuanced, domain-specific dimensions of growth. By reducing the original multidimensional PTGI structure to only two indicators per domain, the PTGI-SF may be less able to detect differentiated associations with external criteria such as affective functioning or quality of life. The possibility that the PTGI-SF captures perceived or illusory growth rather than changes directly reflected in well-being or quality of life should also be considered. This interpretation is consistent with literature suggesting that self-reported PTG may include both constructive and self-enhancing components, and that perceived growth does not necessarily correspond to concurrent improvements in psychological adjustment (16, 44). Accordingly, the non-significant correlations should not be interpreted solely as evidence of theoretical independence between PTG and affective functioning or quality of life. Rather, they may reflect both the conceptual distinctiveness of perceived PTG and limitations of the short-form/unidimensional measurement approach in this specific clinical and linguistic context. Importantly, quality of life in oncology reflects not only emotional well-being but also physical functioning, symptom burden, and treatment-related side effects, which may not improve in parallel with perceived psychological growth (17, 42, 44**).** If post-traumatic growth functions, at least in part, as a cognitive coping or meaning-making process, it may help individuals reinterpret their experience without necessarily translating into measurable and/or immediate improvements in health-related quality-of-life indicators. Therefore, the present findings provide stronger support for the reliability and factorial adequacy of the PTGI-SF total score than for its convergent or divergent validity in this sample. Analyses of temporal stability, however, supported reliability of the PTGI-SF. Test–retest reliability indices were particularly strong (ICC = 0.91), indicating that participants’ perceptions of growth were stable over time at 12- and 18-months after diagnosis, a pattern that has also been reported in community and clinical samples using both the PTGI and its short form (16, 17, 29).

In line with the test–retest findings, the additional comparison of total scores showed no significant changes in either PCL-5 or PTGI-SF scores between the 12- and 18-month assessments. These results suggest stability of self-reported PTS symptoms and perceived PTG over this six-month interval in the present sample. However, this stability should be interpreted cautiously. Although Cordova et al. (11) suggested that trauma-related symptoms may persist after breast cancer treatment, that study was not longitudinal and was based on a relatively small sample (N = 65). Broader person-centered literature indicates that PTS responses after cancer-related experiences may follow heterogeneous trajectories, including stable-low or resilient, recovery, chronic, and worsening patterns (59). Accordingly, the stability observed in the present study should be interpreted as specific to this relatively low-symptom, early-stage breast cancer cohort, rather than as evidence of a uniform trajectory of PTS symptoms or PTG across oncology populations.

The coexistence of PTG with ongoing distress, frequently observed in populations with chronic illnesses, supports the Janus-Face model of PTG, which theorizes that growth comprises both constructive and illusory elements, and may develop independently of symptom reduction (16). It is noteworthy that the present study did not examine the direct relationship between PCL-5 and PTGI-SF scores. This deliberate omission reflects the primary aim of establishing independent psychometric validation for each instrument within this specific population, rather than assuming *a priori* their interdependence. Although theoretical models and prior research support the co-occurrence of PTS and PTG (18), we decided to demonstrate that each instrument functions reliably and validly in isolation, rather than examining their interplay. Furthermore, the temporal dynamics of these constructs may be complex: PTS symptoms typically fluctuate during active treatment, whereas PTG may consolidate only after sufficient time for cognitive processing has elapsed (16). Future longitudinal research should therefore examine how these constructs co-evolve across different phases of the cancer trajectory, using designs to disentangle their temporal precedence and potential reciprocal influences.

The relatively low levels of post-traumatic stress symptoms observed in this sample, with the majority of PCL-5 items showing marked positive skewness, warrant consideration of potential protective factors. No *a priori* hypotheses were established regarding baseline skewness, floor effects, or restricted variability for either the PCL-5 or the PTGI-SF. Rather, these distributional patterns emerged from the descriptive inspection of item-level responses and were used to inform the interpretation of the confirmatory psychometric analyses. Several characteristics of our sample may explain this pattern. First, participants had early-stage breast cancer (stages I-III), for which medical prognosis is generally favorable, potentially attenuating perceived life threat compared with advanced disease (7). Second, assessments occurred at 12 and 18 months post-diagnosis, by which time initial shock may have subsided and adaptation processes consolidated for many women (11). Third, the requirement of eligibility for systemic treatment may have selected for patients with better baseline health status and functional capacity. Additionally, unmeasured protective factors, such as social support, dispositional optimism, or meaning-making processes, may have buffered against trauma responses in this specific cohort (15). These considerations suggest that the present findings may best generalize to relatively resilient subgroups of breast cancer patients, and that future research should explicitly examine moderators distinguishing those who experience significant distress from those who demonstrate resilience or growth without concurrent symptomatology. Accordingly, the psychometric performance observed here may not necessarily generalize to samples with higher PTS symptom severity or distributions skewed toward clinically elevated symptom levels. For the PTGI-SF, item responses did not show the same marked floor-effect pattern observed for the PCL-5. However, the homogeneous clinical profile of the sample and the use of a brief 10-item measure may still have limited the instrument’s sensitivity to capture more nuanced or domain-specific aspects of PTG. Thus, although the PTGI-SF showed adequate reliability and factorial performance as a global measure of perceived growth, further studies in larger and more clinically diverse oncology samples are needed to clarify its dimensionality, external validity, and sensitivity to specific PTG domains.

While our work provides valuable information regarding use of instruments to assess PTS symptoms and PTG in patients with cancer, some limitations deserve consideration. Importantly, the moderate sample size and the restriction to women with early-stage breast cancer represent a trade-off between specificity and generalizability. Although this homogeneity may limit the broader applicability of these results across diverse cancer populations, it simultaneously offers notable methodological advantages. By focusing on a specific cancer type and disease stage, we reduced potential sources of heterogeneity related to treatment regimens, prognosis uncertainty, and gender-specific psychosocial challenges, thereby enhancing the internal validity of our findings and enabling more precise characterization of trauma and growth processes in this context. Future research should aim to replicate these findings in larger and more diverse samples, extend longitudinal follow-up beyond 18 months, and explore moderators such as sociodemographic variables, coping strategies and medical factors, including patients with higher PTS symptom severity. Future studies should also include additional external validation measures for the PTGI-SF, drawing on established measures of related but distinct constructs. These may include the PTGI to examine convergence with the original instrument, PTS symptoms assessed with the PCL-5, sense of coherence assessed with the SOC-13, perceived social support assessed with the mMOS-SS, family communication and coping assessed with the F.A.R.E., resilience assessed with the CD-RISC, fear of cancer recurrence assessed with the FCRI-SF, psychological distress assessed with the HADS, affective adjustment assessed with the PANAS, and oncology-specific quality of life assessed with the EORTC QLQ-BR23. The inclusion of these external criteria would allow a more comprehensive evaluation of whether the PTGI-SF captures perceived global growth, specific PTG domains, or related but distinct adaptive processes in women with breast cancer. In conclusion, our findings offer strong support for the psychometric adequacy of the PCL-5 and, to a somewhat more limited extent, the PTGI-SF, in women with breast cancer. In addition to expanding the availability of validated instruments for Portuguese oncological populations, these results support the integrated use of the PCL-5 and PTGI-SF in psycho-oncology, as they capture distinct yet complementary features of the emotional response to cancer. This approach is consistent with international recommendations from professional oncology societies, which emphasize the need for comprehensive psychosocial assessment beyond depression and anxiety (39). Indeed, by demonstrating that PTS symptoms and posttraumatic growth represent coexisting yet distinct aspects of the psychological response to cancer, this research underscores the importance of multidimensional assessment strategies that capture both vulnerability and resilience.

## Data Availability

The raw data supporting the conclusions of this article will be made available by the authors, without undue reservation.
